# Investigation of *N*-Acetyllactosamine and *N*,*N*-Diacetyllactosamine Residues of Seminal Plasma Prolactin-Induced Protein as Ligands Recognized by Galectin-3

**DOI:** 10.3390/ijms252413432

**Published:** 2024-12-15

**Authors:** Anna Kałuża, Katarzyna Trzęsicka, Damian Drzyzga, Mirosława Ferens-Sieczkowska

**Affiliations:** 1 Department of Biochemistry and Immunochemistry, Division of Chemistry and Immunochemistry, Wroclaw Medical University, M. Skłodowskiej-Curie 48/50, 50-369 Wroclaw, Poland; 2 INVICTA, Research and Development Center, Polna 64, 81-740 Sopot, Poland

**Keywords:** galectin-3, prolactin-induced protein, carbohydrate protein interaction, *N*-acetyllactosamine, *N*,*N*-diacetyllactosamine, immunomodulatory epitopes, seminal plasma

## Abstract

Prolactin induced-protein (PIP) has been found to be rich in immunomodulatory epitopes, including *N*-acetyllactosamine (LacNAc) and *N*,*N*-diacetyllactosamine (LacdiNAc) residues, which may constitute ligands for galecin-3 (Gal-3). In the current study, we aimed to investigate the reactivity of galactose- and *N*-acetylgalactosamine-specific lectins with human seminal plasma PIP. Subsequently, we examined the direct interaction between seminal plasma PIP and galectin-3, and next analyzed whether there are any differences in the interaction associated with impaired semen parameters. The reactivity of terminal galactose-presenting glycans in seminal plasma PIP with *Ricinus communis* agglutinin I in the asthenozoospermic group was significantly higher compared to the normozoospermic fertile subjects. Investigating the reactivity of *Wisteria floribunda* lectin with PIP glycans, we found likewise significantly higher relative reactivity in the normozoospermic infertile as well as the oligoasthenozoopermic group compared to the control group. These results are related to the expression of LacdiNAc epitopes in the oligosaccharide chain of PIP. Finally, we observed that PIP reactivity with *Wisteria floribunda* lectin correlates positively with the interaction between galectin-3 and PIP in the seminal plasma. This can suggest that LacdiNAc residues are engaged in the interaction between PIP and galectin-3.

## 1. Introduction

Seminal plasma glycoproteins bear a variety of unique immunomodulatory epitopes, including the following ones containing *β*-galactose: *N*-acetyllactosamine (LacNAc; Gal*β*1-4GlcNdAc) and *N,N*-diacetyllactosamine (LacdiNAc; GalNAc*β*1-4GlcNAc). Such motifs may function as ligands for endogenous *β*-galactoside-recognizing lectins, mainly belonging to the group of galectins. In former research we have identified some seminal plasma glycoproteins rich in terminal galactose or *N*-acetylgalactosamine, including prolactin-induced protein (PIP), semenogelin 1, semenogelin 2, lactotransferrin, fibronectin, and prostate-specific antigen [1]. Among them, PIP seems especially interesting, as it participates in multiple processes in biological systems, including fertility, immunoregulation, antimicrobial activity, tumor progression, and apoptosis [2].

Prolactin-induced protein is also known as gross cystic disease fluid protein (GCDFP-15). It is a small glycoprotein (17 kDa) secreted by various exocrine glands, including seminal vesicles, salivary glands, and sweat glands [3]. It has been postulated to bind numerous molecules of immunoregulatory potential, such as immunoglobulin G (IgG), human zinc-alpha-2-glycoprotein (ZAG), and actin, as well as some others: fibronectin, keratin, and myosin [4,5,6,7]. An important role of PIP in the reproductive process has also been reported. Low concentration of this protein in human seminal plasma was found to be associated with male infertility [8,9], while formation of zinc-alpha-2-glycoprotein-PIP complex was associated with reduced sperm motility [6]. Moreover, PIP also binds to the CD4^+^ molecule on T cells. High affinity of this interaction suggests that PIP may be involved in the modulation the immune response during the insemination process [2,4,5,10].

PIP has already been reported to be a glycoprotein ligand for galectin-3 (Gal-3) in human prostasomes, together with Mac-2 binding protein (M2BP) and semenogelins 1 and 2 [11]. Galectin-3 is present in semen, secreted by the testis, epididymis, and extracellular vesicles. It is also present on the surface of human sperm [12]. Recently, it has been shown that galectin-3 mediates the binding of human spermatozoa to the zona pellucida and affects fertilization in vitro [13]. The role of seminal plasma galectin-3 in sperm function and fertilization is still not known. Galectin-3 belongs to the chimera-type galectin family with a conserved C-terminal carbohydrate recognition domain (CRD) and an *N*-terminal non-lectin domain [14,15]. Multivalent presentation of ligands plays a significant role in efficient Gal-3 binding, as galectin assembles into pentamers or oligomers upon binding to its oligosaccharide ligands [14,16,17]. As with the other galectins, Gal-3 is classified as a *β*-galctoside-binding lectin. Apart of single-terminal galactose, it is able to recognize and bind *N*-acetyllactosamine and oligo-*N*-acetyllactosamine (oligo-LacNAc) repeating sequences as well as *N*,*N*-diacetyllactosamine disaccharide [14,18]. The LacdiNAc group is rarely observed in mammals, considered to be tissue specific and associated with tumor progression [19,20,21]. Interestingly, the latter glycoepitope recognized by galectin-3 cannot be bound by galectin-1 [22,23,24].

Immunomodulatory protein–carbohydrate interactions may proceed in different directions. Thus, it seems possible that seminal plasma PIP (SP-PIP), as a potential Gal-3 ligand, could participate in postcoital inflammation interactions, as one of the ligands of Gal-3 secreted by vaginal epithelial cells. Although the participation of this lectin in postcoital inflammation has not been described, the secretion and immunomodulatory action of Gal-3 have been described in inflammation associated with vaginal infection [25].

In the current research, we aimed to evaluate the ability of human seminal plasma PIP to bind to galactose-specific lectins, considering possible differences associated with impaired semen parameters of the male subjects. To examine this goal, first we developed ELISA assays using galactose-binding plant lectins, which differ in their preferences towards recognized oligosaccharide structures. The chosen lectins comprised *Ricinus communis* agglutinin I (RCA I), which binds terminal galactose with no special demands, and *Datura stramonium* lectin (DSL), which prefers oligo-LacNAc sequences and *Wisteria floribunda* lectin (WFL), with a special preference for LacdiNAc disaccharide [26], capable of binding terminal galactose or *N*-acetylgalactosamine-containing glycans. Later, we examined the direct interaction of seminal plasma PIP with Gal-3 and analyzed whether there are any differences in the interaction according to the studied groups of men.

## 2. Results

### 2.1. Concentration of Prolactin-Induced Protein in Seminal Plasma

**Figure 1:**
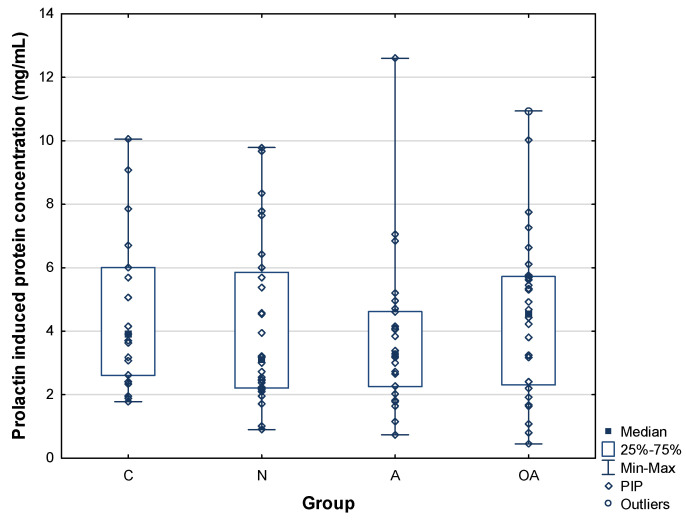
Concentration of prolactin-induced protein (PIP) in seminal plasma. C—control group, N—normozoospermic group, A—asthenozoospermic group, OA—oligoasthenozoospermic group.

The concentration of prolactin-induced protein was quantified with ELISA assay for all patients (n= 83) and control subjects (n = 21). The mean concentration of PIP was similar in all patient groups, namely, 4.15 ± 2.59 mg/mL for the normozoospermic, 3.74 ± 2.34 mg/mL for the asthenozoospermic, and 4.53 ± 2.59 mg/mL for the oligoasthenozoospermic group, compared to 4.71 ± 2.49 mg/mL in the control group (Table 1), without any statistically significant differences. The distribution of PIP values is shown in whisker-box plots, also indicating no difference among the groups (Figure 1). The obtained results are consistent with the previously reported data on PIP concentration, i.e., 2.0–4.4 mg/mL [5].

### 2.2. PIP Reractivity with Galactose-Binding Lectins

In order to screen the presence of terminal galactose or *N*-acetylgalactosamine-containing glycans of the prolactin-induced protein in seminal plasma, we carried out ELISA assays based on the different carbohydrate specificity of the chosen lectins: *Ricinus communis* agglutinin I, *Datura stramonium*, and *Wisteria floribunda*, which prefer all *β*-galactosides or selectively binds oligo-LacNAc and LacdiNAc residues, respectively.

#### 2.2.1. Ricinus Communis Agglutinin I Reactivity

**Figure 2:**
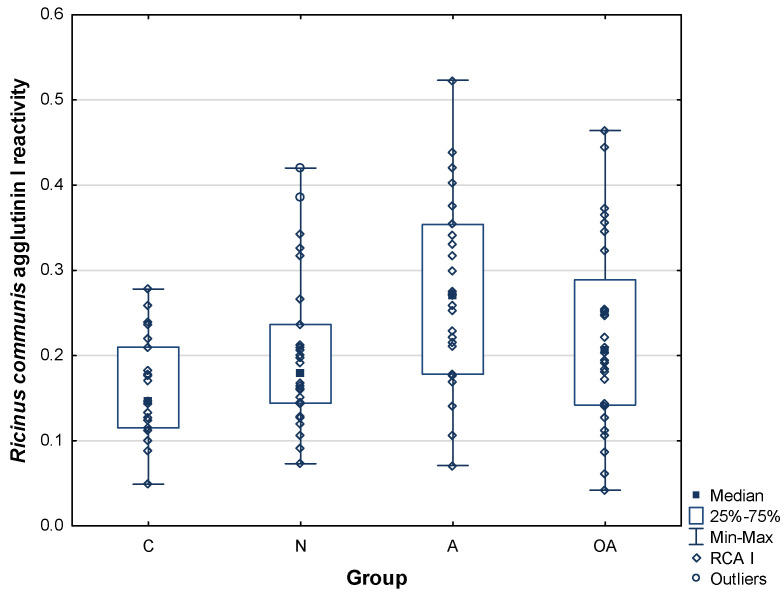
Relative reactivity of PIP glycans with *Ricinus communis* agglutinin I (RCA I). C—control group, N—normozoospermic group, A—asthenozoospermic group, OA—oligoasthenozoospermic group.

PIP reactivity with RCA I, which binds terminal galactose residues without any further preference, is presented in Table 2, and the distribution of values is presented in Figure 2. Mean and median values were higher in infertile subjects compared to the control group (0.20 ± 0.09 for N, 0.27 ± 0.11 for A, and 0.22 ± 0.11 for OA vs. 0.16 ± 0.06 AU for C). In asthenozoospermic subjects, this increase reached a statistically significant level (*p*^C^ = 0.002140).

#### 2.2.2. Datura Stramonium Lectin Reactivity

**Figure 3:**
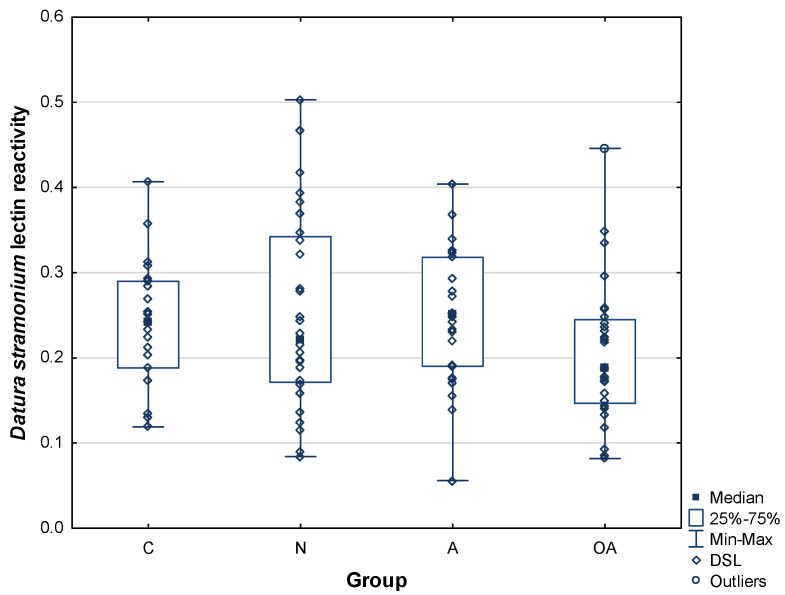
Relative reactivity of PIP with *Datura stramonium* lectin (DSL). C—control group, N—normozoospermic group, A—asthenozoospermic group, OA—oligoasthenozoospermic group.

*Datura stramonium* lectin prefers *N*-acetyllactosamine repeats in galactose-containing oligosaccharides. Looking at its binding to human seminal plasma PIP, we did not find any differences among the studied groups in either mean values or in value distribution (Table 2 and Figure 3). The mean values for the analyzed groups were found to be as follows: 0.24 ± 0.07 AU for the control group, 0.25 ± 0.11 AU for the normozoospermic group, 0.25 ± 0.08 AU for the asthenozoospermic group, and 0.20 ± 0.08 AU for the oligoasthenozoospermic group (Table 2).

#### 2.2.3. Wisteria Floribunda Lectin Reactivity

**Figure 4:**
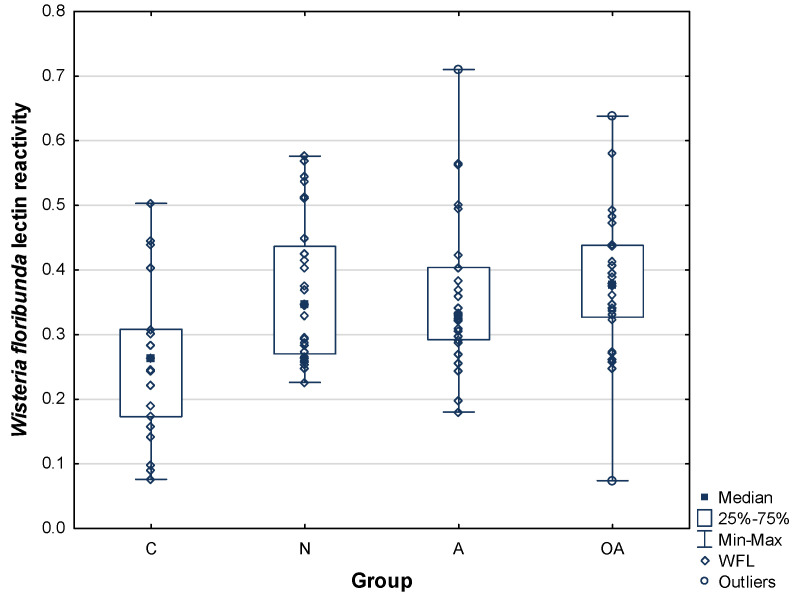
Relative reactivity of PIP glycans with *Wisteria floribunda* lectin (WFL). C—control group, N—normozoospermic group, A—asthenozoospermic group, OA—oligoasthenozoospermic group.

**Figure 5:**
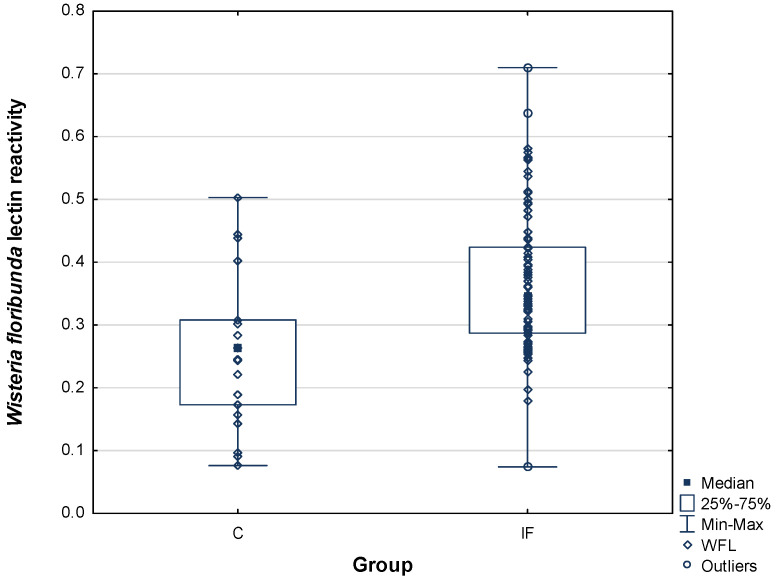
Relative reactivity of PIP glycans with *Wisteria floribunda* lectin (WFL) for the control group (C) and the infertile group (IF), which contained all infertile subjects gathered together.

*Wisteria floribunda* is a lectin with especially interesting specificity. It prefers the so-called LacdiNAc group, the lactose derivative in which both components, galactose and glucose, are *N*-amino-acetylated at C2. When *Wisteria floribunda* lectin reactivity with PIP glycans was studied, once again we observed increased levels of binding in infertile vs. control subjects (Table 2 and Figure 4). Relative reactivity of WFL with PIP glycans in seminal plasma of the fertile subjects was 0.26 ± 0.12 AU compared to 0.37 ± 0.11 AU for the normozoospermic infertile group, 0.36 ± 0.12 AU for the asthenozoospermic group, and 0.38 ± 0.11 AU for the oligoasthenozoospermic group. While in the asthenozoospermic subjects the slight increase in PIP-lectin reactivity did not reach statistical significance, in the normozoospermic and oligoasthenozoospermic groups the increase was significant, at *p*^C^ *=* 0.025316 and *p*^C^ *=* 0.002515, respectively (Table 2). However, the increase in reactivity remained statistically significant also when all the infertile subjects were gathered into one group *p*^C^ = 0.000367 (Figure 5).

### 2.3. Galectin-3 Reactivity with SP-PIP

**Figure 6:**
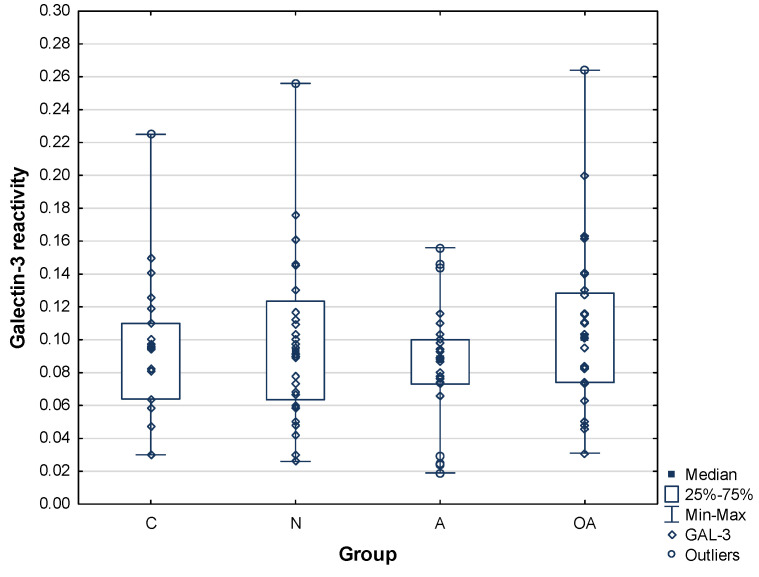
Relative reactivity of prolactin-induced protein glycans with galectin-3. C—control group, N—normozoospermic group, A—asthenozoospermic group, OA—oligoasthenozoospermic group.

Galectin-3 relative reactivities with seminal PIP were 0.096 ± 0.04 AU for the control group, 0.099 ± 0.05 AU for the normozoospermic group, 0.085 ± 0.03AU for the asthenozoospermic group, and 0.11 ± 0.05 AU for the oligoasthenozoospermic group. Contrary to plant lectins, PIP reactivity with Gal-3 did not show any difference among the studied groups of subjects. The mean values are summarized in Table 3, and the data distribution is shown in Figure 6.

### 2.4. Spearman Rank Correlation Test

**Figure 7:**
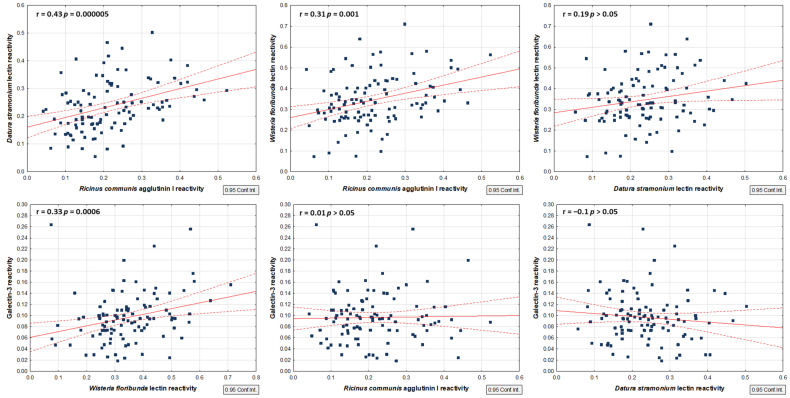
Correlation scatter plots of PIP reactivity between the studied lectins: *Ricinus communis* agglutinin I (RCA I), *Datura stramonium* lectin (DSL), and *Wisteria floribunda* lectin (WFL), and galectin-3. The dark blue squares represent the set of points. The solid red line is the line of best fit - the trend line, while the dashed red line indicates the 95% confidence interval.

Galectin-3, like the other lectins from this group, recognizes *β*-galactose residues occupying the terminal position in the oligosaccharide chain. It can be assumed that, similarly to other lectins, Gal-3 may show some preferences for more precisely defined structures, but there is no such information to date. Therefore, we decided to compare Gal-3–PIP reactivity with the reactivity of plant lectins, whose preferences are better defined. We examined the correlations in the PIP reactivity of plant lectins among each other, as well as the correlations between PIP reactivity with each of the plant lectins and with that of galectin-3. The results of these analyses are presented in Figure 7.

In two out of three pairs of plant lectins, a positive correlation was observed: a moderate one (r = 0.43; *p* = 0.000005) in the case of RCA I relative reactivity vs. DSL, and a weak one (r = 0.31, *p* = 0.001) regarding RCA I vs. WFL (Table 4). These results point to the presence of galactose corresponding to oligo-LacNAc epitopes and LacdiNAc motifs. It also shows that an involvement of *β*-galactose in the formation of a LacNAc or LacdiNAc structure does not influence general *β*-galactose–RCA I binding. The lack of a statistically significant correlation between DSL and WFL confirms that both lectins prefer distinct, although partially overlapping, carbohydrate epitopes.

Comparing the reactivity of plant lectins and Gal-3, a statistically significant positive correlation was observed only in the case of the *Wisteria floribunda* lectin. Interestingly, the reactivity of this lectin was significantly increased in two out of three groups of infertile men, as well as in the group of infertile men gathered together (Figure 4 and Figure 5). These results may suggest that the presence of the LacdiNAc glycoepitope may be beneficial for PIP–Gal-3 binding, especially in the seminal plasma of infertile men. Although with the other lectins no statistically significant correlations were shown, in the case of *Datura stramonium* lectin we observed a slight but noticeable tendency towards a negative correlation (Figure 7). This in turn may suggest that extension of PIP glycans with LacNAc repeats may weaken Gal-3 interaction.

## 3. Discussion

The oligosaccharide structures of glycoproteins show enormous diversity. Lectins, proteins with a carbohydrate-recognition domain, often have the ability to distinguish even small structural differences in oligosaccharides. Such a difference can facilitate or hinder, sometimes even preventing binding and changing its kinetics. Lectins acting in the human immune system have their carbohydrate-recognition domains connected to those responsible for other activities—for example, initiating metabolic pathways or protein synthesis. Therefore, a small structural difference in the oligosaccharide chain may be important for modulating other lectin-mediated activities [27].

Protein glycosylation is crucial for spermatozoa present in seminal plasma and in the later stages of fertilization, from conception and implantation to childbirth. Especially significant are antennae sequences of *N*- and *O*-glycans, which are potential ligands for endogenous lectins. A wide repertoire of glycan epitopes is known to be expressed in human male and female reproductive tracts, where many glycoconjugates participate in sperm development and promote sperm migration and survival in the female reproductive system [28,29,30,31]. In our previous review, we hypothesized that the presence of immunosuppressive glycans on the sperm surface and in seminal plasma glycoproteins might contribute to improved fertilization outcomes [32]. Also, former studies have attempted to identify glycoproteins that can bear such immunomodulatory glycans. Among them, prolactin-induced protein, lactotransferrin, fibronectin, semenogelin 1 and 2, and prostate specific antigen have been reported as being rich in terminal galactose and LacNAc and LacdiNAc motifs; thus, they could be potential ligands for *β*-galactose binding proteins such as galectin-3 [1]. Xin et al. revealed highly expressed LacNAc and LacdiNAc structures in acrosomal protein, zona pellucida-binding protein 1 and 2, Izumo sperm–egg fusion protein, equatorin and acrosin-binding protein [33]. Potential galectin-3 ligands in seminal plasma prostasomes, including Mac-binding protein-2, dipeptidyl peptidase IV, prolactin-induced protein, olfactomedin-4, and semenogelins 1 and 2, were reported by Block et al. [11]. Also, Kovak et al. identified prostate-specific antigen, prostatic acid phosphatase, zinc alpha-2-glycoprotein (ZAG), dipeptidyl peptidase-4, aminopeptidase N, neprilysin, clusterin, antibacterial protein, and alpha-1-acid glycoprotein as galectin-3 binding proteins by means of affinity column chromatography [34,35]. Basing on these data, in the current study we decided to analyze SP-PIP as a potential carrier of glycans containing galactose and its derivatives.

To establish the analytical model, we had to determine PIP concentration in seminal plasma samples. Although some reports indicated a decrease in PIP concentration in the SP of asthenozoospermic men [36,37], in our study the protein concentration was similar in all examined groups, in accordance with the data reported by Chiu et al. [5]. Quantitative determination of PIP concentration was used to establish the conditions for further analyses of the protein reactivity with lectins.

Galectins belong to the group of lectins recognizing *β*-galactoside structures. As for a more precise definition of their specificity, or rather, preferences, little is known, and such information may be important for the activity resulting from sugar attachment. The current study confirmed that human SP-PIP is recognized by galectin 3. Analysis of the direct reaction of PIP with Gal-3 did not show statistically significant differences between the analyzed groups of fertile men and those with reduced fertility. Nevertheless, a detailed analysis of the reactivity of the protein with plant lectins of well-defined specificity showed that certain structural differences in PIP glycans are associated with infertility, and regardless of the type of abnormalities observed in semen related to sperm count or motility.

We used three plant lectins differing in the binding preferences as tools and further checked the direct interaction of the glycoprotein with Gal-3. Though SP-PIP reactivity with RCA I is not surprising, subsequent binding of DSL and WFL shows that the glycoprotein also bears less common glycoepitopes like LacNAc repeats and LacdiNAc. The latter was associated with the infertility status of the examined subjects.

The most interesting observation in the current study is that the reactivity of PIP glycans with WFL correlated positively with the reactivity of PIP and Gal-3. PIP bound more strongly to *Wisteria floribunda* lectin when it contained more LacdiNAc structures, and the PIP that reacted more strongly with *Wisteria floribunda* lectin also bound to Gal-3 to a higher extent, as evidenced by the positive correlation. This may suggest that the PIP–Gal-3 interaction is more intense in the presence of LacdiNAc structures. On this basis, it can be suggested that Gal-3 shows a certain preference for LacdiNAc structures present in SP-PIP, though this is only indirect speculation.

Another question that arises is whether this fact may be related to reduced male fertility. Since this may be hypothesized based on our results, confirmation of such a hypothesis demands further, more detailed research, including glycan structure analysis as well as kinetic studies. While the study highlights the promising outcomes, it also has some limitations, namely, the modest size of the investigated groups. Therefore, further validation in a larger cohort of patient samples is essential to confirm the reliability of the findings and enhance the robustness of the conclusions.

## 4. Materials and Methods

### 4.1. Seminal Plasma Samples

Seminal plasma samples were collected from patients of the INVICTA Fertility Clinic in Wrocław. Semen samples were obtained by masturbation after 2–3 days of sexual abstinence and liquefied at 37 °C within an hour of collection. Standard semen analysis was performed according to World Health Organization directives (WHO 2021), resulting in gathering the samples into the following groups: normozoospermic (N; n = 28; semen parameters within the WHO’s normal range), asthenozoospermic (A; n = 27; total sperm motility < 42%), and oligoasthenozoospermic (OA; n = 28; spermatozoa count < 16 mln/mL and total sperm motility < 42%). As the control group (C), 21 healthy human volunteers were recruited, and their semen samples were obtained and processed in the same way. All the control group subjects had semen parameters within the WHO’s normal range, and all of them had fathered children. The remaining semen samples were gently centrifuged at 400 x *g* for 10 min at room temperature to remove spermatozoa, and the seminal plasma obtained this way was aliquoted and stored at −80 °C until use. The normozoospermic group included men living in childless couples, who attended the clinic with their partners for infertility treatment. Thus, the exclusion factor was an evident female factor of infertility, such as ovulation disorders, blocked fallopian tubes, or endometriosis. In the presented normozoospermic group, we were dealing with idiopathic infertility, unrelated to disorders in the semen pattern. The subjects who qualified for the study did not include heavy drinkers or excessive smokers.

All study participants provided written informed consent, and the study was approved by Wrocław Medical University Bioethics Council (approval number KB-330/2023N).

### 4.2. Quantification of Prolactin-Induced Protein

Prolactin-induced protein in seminal plasma samples was determined using the Human GCDFP 15 ELISA Kit from Abcam (ab284017, Abcam, Cambridge, UK) according to the manufacturer’s instructions. Commercially prepared anti-h-PIP antibody-coated microtiter plates were loaded with 50 µL of PIP standard (0.625–20.0 ng/mL) and 10^6^-fold diluted seminal plasma samples. After 2 h of incubation at RT, the plate was extensively washed with PBS-T (0.05% Tween 20 in PBS; 10 mM phosphate buffer, pH 7.4, containing 0.9% NaCl). Next, the biotinylated human anti-GCDFP 15 antibodies, diluted 50-fold, were used as a detection antibody and incubated for one hour at room temperature. After another extensive washing, 50 µL of streptavidin–horseradish peroxidase conjugate solution (Streptavidin-HRP), diluted 100-fold, was applied to each plate well. After 30 min of incubation and another washing, the enzymatic reaction was developed with TMB (3,3′-5,5′-tetramethylbenzidine) solution. After 20 min, the reaction was stopped with 1 M H_2_SO_4_ and the absorbance was read with a Synergy LX Multi-Mode Reader (BioTek Instruments, Inc., Winooski, VT, USA) at a wavelength of 450 nm with a reference filter of 570 nm. All seminal plasma samples were assayed in duplicate, and the mean value of two replicates was used for statistical analysis. The intra- and inter-assay coefficients of variation (CV%) for PIP concentration were calculated as CV% 4.4 and CV% 8.6, respectively.

### 4.3. Evaluation of LacNAc and LacdiNAc Residues of Seminal Plasma PIP

The initial assay setup was performed using two different types of coating agent: monoclonal (ab218480, Abcam, Cambridge, UK) or polyclonal (RD181304100, Biovendor, Brno, Czech Republic) antibodies. Finally, a 96-well microtiter plate (Nunc MaxiSorp, Thermo Fisher Scientific, Waltham, MA, USA) was coated with 100 µL of mouse anti-human prolactin-induced protein monoclonal antibodies (anti-h-PIP, ab218480, Abcam, Cambridge, UK), applied in a concentration of 1 µg/mL and incubated for two hours at 37 °C due to the low absorbance value of the blank sample in this case. Afterwards, the plate was washed five times with 10 mM PBS, pH 7.4, and free binding sites were blocked with 1% BSA in 10 mM PBS for two hours at 37 °C. Seminal plasma samples were diluted in 10 mM PBS, pH 7.4 buffer, to obtain 1μg/100 μL PIP concentration per well, applied to plate wells in duplicate, and incubated for one hour at 37 °C. Next, after extensive washing with PBS-T, the biotinylated *Ricinus communis* agglutinin I (RCA I, 1 µg/mL), *Datura stramonium* (DSL, 1 µg/mL), or *Wisteria floribunda* (WFL, 2 µg/mL) lectin were added, and the samples were incubated for one hour at 37 °C. Lectin binding was detected using ExtrAvidin-AP (ExtrAvidin-alkaline phosphatase labeled), diluted 10,000-fold, and incubated for thirty minutes at 37 °C. The reaction was developed with disodium *para*-nitrophenyl phosphatase substrate (*p*-NPP, 1.0 mg/mL), and after twenty minutes the reaction was quenched with 1 M NaOH (100 μL/well). The absorbance values were measured with a Synergy LX Multi-Mode Reader (BioTek Instruments, Inc., Winooski, VT, USA) at a wavelength of 405 nm with a reference filter of 630 nm. The final reactivity values of the selected lectins were expressed in arbitrary units (AU) defined as the absorbance presented by 1 µg of PIP in the method conditions, after subtracting the absorbance of the blank sample. Glycophorin was used as a positive control to confirm *Ricinus communis* agglutinin I, *Datura stramonium*, and *Wisteria floribunda* lectin binding, while yeast carboxipeptidase Y (Sigma-Aldrich, St. Louis, MO, USA) was included as a negative control.

### 4.4. Evaluation of Prolactin-Induced Protein Interaction with Galectin-3

An ELISA microplate was coated with 100 µL of monoclonal anti-h-PIP antibodies (ab218480, Abcam, Cambridge, UK), applied in a 1 µg/mL concentration, and incubated for two hours at 37 °C. After washing, free binding sites on the plate were blocked with 1% BSA in PBS for two hours at 37 °C. In the next step, seminal plasma samples were diluted to obtain 5 μg of PIP per well. Samples (100 µL) were applied to plate wells in duplicate and incubated for one hour at 37 °C with gentle shaking. Next, after washing with PBS-T, the plate was incubated with human galectin-3 (100 µL, 1 µg/mL, ab89487, Abcam, Cambridge, UK) and incubated for one hour at 37 °C. After the next extensive washing, biotinylated anti-h-galectin-3 antibodies (0.5 µg/mL, ab84238, Abcam, Cambridge, UK) were used as detection antibodies, and the plate was incubated for 30 min at 37 °C. Following the next washing, 100 µL of ExtrAvidin-AP, diluted 10,000-fold, was applied to each well and incubated for 30 min at 37 °C. A color reaction was developed with 100 µL of *p*-NPP (1.0 mg/mL), and after twenty minutes it was quenched with 100 µL of 1 M NaOH. Absorbance values were read on a Synergy LX Multi-Mode Reader (BioTek Instruments, Inc., VT, USA) at a wavelength of 405 nm with a reference filter of 630 nm. The final absorbance values are shown in the arbitrary unit after subtracting the absorbance of the blank sample, as defined in former settings.

### 4.5. Statistical Analysis

Statistical analysis was performed with Statistica 13.3 software (StatSoft, Inc., Tulsa, OK, USA). Prolactin-induced protein concentration and PIP relative reactivities with the investigated lectins are presented as mean ± SD and illustrated in the box-whisker plots, with the median and 25th–75th percentiles indicated. The Mann–Whitney U test was applied to compare PIP concentration, its reactivity with plant lectins, and its reactivity with Gal-3 among the examined groups. A *p*-value lower than 0.05 was considered statistically significant. Normality of data distribution was analyzed by means of the Shapiro–Wilk test. Scatter plots representing the correlations between the relative reactivity of PIP glycans with *Ricinus communis* agglutinin I, *Datura stramonium*, or *Wisteria floribunda* lectins, as well as with Gal-3, were prepared based on Spearman’s rank correlation test for all subjects.

## Figures and Tables

**Table 1:** PIP concentration in seminal plasma of examined subject groups.

PIP Concentration (mg/mL)	Group				
		C	N	A	OA
		*n =* 21	*n =* 28	*n =* 27	*n =* 28
	Mean ± SD	4.71 ± 2.49	4.15 ± 2.59	3.74 ± 2.34	4.53 ± 2.59
	Range	1.78–10.05	0.09–9.79	0.74–12.60	0.45–10.94

C—control group, N—normozoospermic group, A—asthenozoospermic group, OA—oligoasthenozoospermic group.

**Table 2:** Relative reactivity of selected plant lectins with human seminal plasma PIP glycans.

Lectins Reactivity (AU)	Group				
		C	N	A	OA
		*n =* 21	*n =* 28	*n =* 27	*n =* 28
RCA I reactivity	Mean ± SD	0.16 ± 0.06	0.20 ± 0.09	0.27 ± 0.11	0.22 ± 0.11
				*p^C^ =* 0.002140	
	Range	0.05–0.28	0.07–0.42	0.07–0.52	0.04–0.46
DSL reactivity	Mean ± SD	0.24 ± 0.07	0.25 ± 0.11	0.25 ± 0.08	0.20 ± 0.08
	Range	0.12–0.41	0.08–0.50	0.06–0.40	0.08–0.45
WFL reactivity	Mean ± SD	0.26 ± 0.12	0.37 ± 0.11	0.36 ± 0.12	0.38 ± 0.11
			*p^C^ =* 0.025316		*p^C^ =* 0.002515
	Range	0.08–0.50	0.23–0.58	0.18–0.71	0.07–0.64

Lectin reactivity is shown in arbitrary units (AU), defined as the absorbance representing the reactivity of 1 µg of PIP, as mean values ± standard deviation (SD). The *p*-values lower than 0.05 were considered statistically significant.

**Table 3:** Galectin-3 reactivity with seminal plasma PIP.

Lectins Reactivity (AU)	Group				
		C	N	A	OA
		*n =* 21	*n =* 28	*n =* 27	*n =* 28
Gal-3 reactivity	Mean ± SD	0.096 ± 0.04	0.099 ± 0.05	0.085 ± 0.03	0.11 ± 0.05
	Range	0.03–0.23	0.03–0.26	0.02–0.16	0.03–0.26

**Table 4:** Results of the Spearman rank correlation test of PIP reactivity between the studied lectins: *Ricinus communis* agglutinin I (RCA I), *Datura stramonium* lectin (DSL), *Wisteria floribunda* lectin (WFL), and galectin-3.

Spearman Rank Correlation Test		
Lectin Relative Reactivity	r	*p*
RCA I vs. DSL	0.43	0.000005
RCA I vs. WFL	0.31	0.001
DSL vs. WFL	0.19	*p* > 0.05 (NS)
Galectin-3 relative reactivity	r	*p*
GAL-3 vs. WFL	0.33	0.0006
GAL-3 vs. RCA I	0.01	*p* > 0.05 (NS)
GAL-3 vs. DSL	−0.1	*p* > 0.05 (NS)

r—Spearman’s rank correlation coefficient; *p*-value lower than 0.05 was considered statistically significant; NS—not significant.

## Data Availability

The data underlying this article will be shared on reasonable request to the corresponding author.
